# Poor hypotheses and research waste in biology: learning from a theory crisis in psychology

**DOI:** 10.1186/s12915-025-02134-w

**Published:** 2025-02-04

**Authors:** Shinichi Nakagawa, David W. Armitage, Tom Froese, Yefeng Yang, Malgorzata Lagisz

**Affiliations:** 1 Department of Biological Sciences, University of Alberta, CW 405, Biological Sciences Building, Edmonton, AB T6G 2E9 Canada; 2 Theoretical Sciences Visiting Program (TSVP), Okinawa Institute of Science and Technology Graduate University, 1919-1 Tancha, Onna, Kunigami District, Okinawa, 904-0412 Japan; 3 Evolution & Ecology Centre and School of Biological, Earth and Environmental Sciences, University of New South Wales, Sydney, NSW 2052 Australia; 4 Integrative Community Ecology Unit, Okinawa Institute of Science and Technology Graduate University (OIST), 1919-1 Tancha, Onna, Okinawa, 904-0495 Japan; 5 Embodied Cognitive Science Unit, Okinawa Institute of Science and Technology Graduate University (OIST), 1919-1 Tancha, Onna, Okinawa, 904-0495 Japan

**Keywords:** Questionable research practice, Replication crisis, Credibility revolution, Team science, SORTEE

## Abstract

While psychologists have extensively discussed the notion of a “theory crisis” arising from vague and incorrect hypotheses, there has been no debate about such a crisis in biology. However, biologists have long discussed communication failures between theoreticians and empiricists. We argue such failure is one aspect of a theory crisis because misapplied and misunderstood theories lead to poor hypotheses and research waste. We review its solutions and compare them with methodology-focused solutions proposed for replication crises. We conclude by discussing how promoting inclusion, diversity, equity, and accessibility (IDEA) in theoretical biology could contribute to ameliorating breakdowns in the theory-empirical cycle.

## Drivers of research waste: is there a theory crisis in biology?

“An approximate answer to the right question is worth a great deal more than a precise answer to the wrong question.” John Tukey

Many scientific fields have faced a *replication crisis* (refer to Glossary in Table 1) due to the low replicability of empirical studies. In biology, this is perhaps most well-known in cancer studies (see, for instance, the “Reproducibility Project: Cancer Biology”; https://elifesciences.org/collections/9b1e83d1/reproducibility-project-cancer-biology), although it arguably first gained significant attention in the social sciences, particularly in psychology [1–4]. In response, recent reforms have changed how psychologists conduct their research [5, 6] (also see [7]). One example is their adoption of *pre-registration* and *registered reports*, which can involve pre-commitment to a particular set of hypotheses and study design, often involving peer review [8–11]. For example, more than 130 psychology journals now accept registered reports in 2020 (compared to six in 2015) [12] (see also [13]). These innovations can curtail *questionable research practices* (QRPs) such as selective reporting and *HARKing* (hypothesizing after results are known), ubiquitous in all scientific fields, including biology [14–18]. In one example, psychological studies based on pre-registered reports supported the author’s hypotheses only 40% of the time, whereas traditional non-pre-registered studies supported the authors’ hypotheses over 85% [19]. Similar reforms are being implemented in biology, particularly ecology and evolutionary biology, though progress is slower [20–22]. We concentrate on ecology and evolutionary biology for the reasons outlined below, although the issues and solutions are relevant to all branches of biology.

**Table 1:** Glossary

**Replication crisis:** A situation where many scientific studies cannot be replicated or reproduced, casting doubt on the reliability of published findings (see below for the definitions of the narrow-sense and broad-sense replication crisis).
**Pre-registration:** the practice of registering study aims, hypotheses, methods, and analysis plans before conducting research to increase transparency and reduce bias (usually without peer review).
**Registered reports:** publishing format where study proposals are peer-reviewed and accepted before data collection begins, emphasizing the importance of the research question and methodology.
**Questionable research practices (QRPs):** Research behaviors that compromise the integrity and validity of scientific findings, such as selectively reporting results or hypothesizing after results are known.
**HARKing: **Acronym for “Hypothesizing After Results are Known”; the practice of formulating hypotheses based on data after the fact and presenting them as if they were established before data collection.
**Research waste:** The inefficient use of resources in research due to issues like unpublished negative results, incomplete reporting, or flawed study design, leading to findings that are unusable or unreliable.
**Incomplete reporting:** Failure to fully disclose research methods, data, or results, hindering replication and the accumulation of scientific knowledge.
**Poor methodological design:** Inadequate or flawed research design, data collection, or analysis that undermines the validity and reliability of study findings.
**Theory crisis:** A situation where a scientific field lacks robust, testable theories or suffers from misinterpretation and misapplication of existing theories, leading to poor hypotheses and research waste.
**Narrow-sense replication crisis (method crisis):** The aspect of the replication crisis attributable specifically to methodological issues, such as poor study design and statistical practices.
**Researcher degrees of freedom: **The flexibility researchers have in making decisions during data collection, analysis, and reporting that can influence study outcomes (also referred to as the garden of forking paths).
**Broad-sense replication crisis: **An expanded view of the replication crisis that encompasses both methodological and epistemological issues (i.e., narrow-sense replication crisis and theory crisis), which acknowledges that difficulties in replicating studies arise not only from flawed methods but also from misapplied or misunderstood theories.
**Theoretical model: **A formal representation of a theory using mathematical, computational, or conceptual frameworks to make precise predictions although understanding such a model can be difficult.
**Null hypothesis significance testing (NHST)**: A dominant statistical framework where researchers test whether there is enough evidence to reject a null hypothesis in favor of an alternative hypothesis.
***P*-hacking (P-value hacking):** Manipulating data or analyses to achieve statistically significant results, often by trying multiple analyses until a desired p-value is obtained (*p*-hacking is also known as data dredging or fishing expedition).
**Cherry-picking (selective reporting):** The practice of selectively reporting only favorable results while ignoring or omitting others, leading to biased conclusions.
**H-BUTing:** Acronym for “Hypothesizing Before Understanding Theory”; formulating hypotheses without fully understanding the underlying theory, which can result in irrelevant or flawed research.
**Hypothesis hacking (*h*-hacking):** Deliberately manipulating or selectively interpreting theories to generate hypotheses that align with preconceived beliefs or desired outcomes.
**Hypothesis cherry-picking:** Intentionally ignoring alternative, theoretically valid hypotheses that do not support one’s initial beliefs or preferred interpretations.
**Strong inference:** A scientific approach involving the development of multiple competing hypotheses and designing experiments to systematically eliminate them, strengthening the validity of conclusions.
**Construct validity:** The extent to which a test or experiment accurately measures the theoretical construct it intends to measure.
**Internal validity:** The degree to which a study establishes a trustworthy cause-and-effect relationship between variables, free from confounding factors.
**External validity:** The extent to which study findings can be generalized beyond the specific context of the research.
**Non-confirmatory studies:** Research conducted without specific hypotheses, aimed at exploring data to generate new hypotheses and insights (also known as exploratory or discovery-oriented studies).
**Hackathon:** An event where individuals collaborate intensively on projects or problems over a short period, fostering creativity and innovation across various fields, not limited to programming.
**Meta-analyses: **Statistical analyses that combine results from multiple studies to identify patterns, assess the robustness of findings, and provide a more precise estimate of effects.
**Systematic reviews:** Comprehensive reviews of literature on a specific topic using systematic and transparent methods to minimize bias and synthesize findings.
**Systematic maps:** Structured collections of research studies that aim to describe the state of knowledge on a topic, identifying gaps and clusters without necessarily synthesizing results.
**Research weaving:** An integrative approach that combines systematic mapping and bibliometrics to visualize and understand the structure and development of research fields.
**IDEA:** Acronym for Inclusion, Diversity, Equity, and Access; principles that promote fair and inclusive practices in academia and research.
**Unconference: **A participant-driven meeting where attendees create the agenda and lead discussions, fostering open exchange of ideas without predefined topics.

**Fig. 1:**
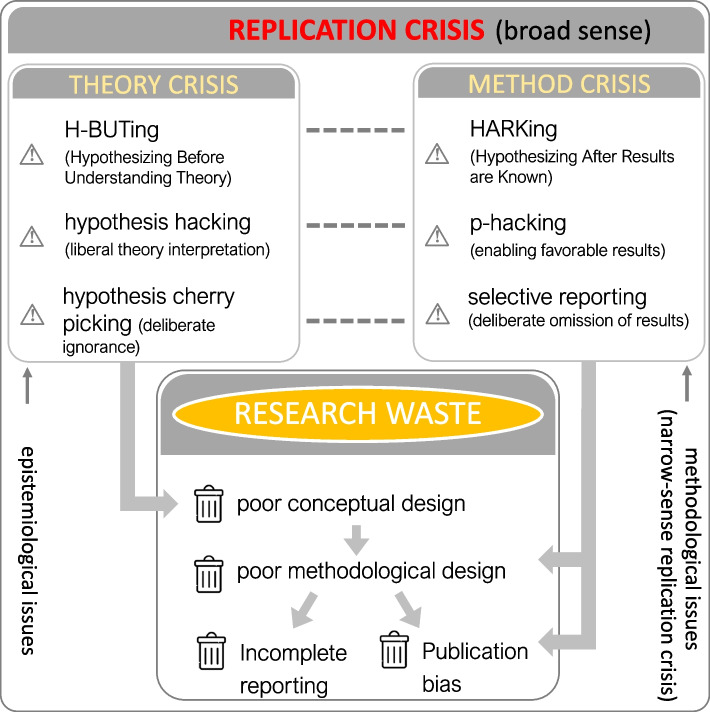
Questionable research practices, QRPs, arising from a theory crisis (via epistemological issues) and method crisis (narrow-sense replication crisis; via methodological issues) and how they relate to research waste. QRPs concerning the theory crisis relate to poor conceptual design, which comes upstream of the other 3 items of research waste. Note that selective reporting and incomplete reporting may sound similar, but the former indicates deliberate selection of positive results while the latter represents the lack of culture in providing all the results and associated outcomes, including associated data and code

The replication crisis, driven by QRPs, is closely tied to *research waste*. According to Purgar and colleagues [23], research waste occurs when scientific resources are expended without yielding reliable, informative results to researchers and other end users. First, scientists complete projects but never publish or terminate them early, often due to negative results, leading to *publication bias*. Second, scientists publish their results with insufficient reporting of methods, resulting in *incomplete reporting*. Third, the research suffers from poor study design, inadequate data collection, or flawed analysis, culminating in *poor methodological design*; even if published, results are unreliable and unusable. Alarmingly, Purgar and colleagues estimated that research waste could be as high as 82–89% in ecology [23], mirroring the estimate of 85% for medical sciences by Chalmers and Glasziou [24] (see also [25–29]). Since both the replication crisis and research waste seem to stem from methodological or procedural shortcomings, reforms have appropriately focused on rigorous and transparent methodology and reporting practices, including open data and code [30–33]. Therefore, it appears that a replication crisis should be more appropriately called a *method crisis* (we also refer to it as a *narrow-sense replication crisis*; see Fig. 1).

However, a series of articles in psychology have highlighted a more fundamental problem contributing to both research waste and replication difficulties—the emergence of a *theory crisis* [34–41]. This theory crisis arises when researchers frequently test vague and incorrect hypotheses because the underlying theories are often verbal (i.e., informal), allowing researchers to interpret them more freely, akin to high *researcher degrees of freedom* [42]. Even when written formally as mathematical models, a theory is often poorly described, leading to misinterpretation by empiricists and a mismatch between theoretical predictions and empirical hypotheses. This situation has been called an interpretation crisis [34] (cf., generalizability crisis [40]). As the opening quote from John Tukey suggests, testing a precise hypothesis originating from a misapplied or misunderstood theory is undoubtedly a form of research waste or, at least, research inefficiency.

Additional signals of a theory crisis include an overall lack of theories (and theoreticians) or the prevalence of poorly reasoned or vague theories. Yet, the lack of formalization and the prevalence of poor interpretations seem to be a substantial part of the discussion on the theory crisis in psychology (cf. [36–38]). Therefore, we use the term a theory crisis to include both (1) a lack of testable, formal theories within a field and (2) misinterpretations of sufficiently developed theories by empirical researchers. These theoretical shortcomings contribute to what we might call the *broad-sense replication crisis*, encompassing both methodological issues (i.e., a narrow-sense replication or method crisis) and fundamental epistemological challenges related to theory development and application (i.e., a theory crisis; Fig. 1).

To our knowledge, theory crises have not been explicitly discussed and linked to research waste in biological research (cf. [21]). Yet there has been much debate about communication gaps and failures between theoreticians and empiricists in biology, especially in ecology and evolution [43–51] (also see [52]; hereafter, we use a *theoretical model* or model as a mathematically formalized version of theory). Such debates indicate a theory crisis, per our definition, may have long existed and remain unresolved in biology. For example, in a 2022 paper [47], Servedio reported survey results from theoreticians in ecology and evolution on how their models are used in empirical work. Models were reported as misinterpreted and used incorrectly 19% of the time, while 36% of the time, they were cited in a non-specific manner. That is, the rest of the time (45%), empiricists cited models correctly, relating their work to specific theoretical predictions. Whether 45% seems low or high, there is much to improve for what both 91% of theoreticians and 80% of empiricists agreed upon—the importance of a tight feedback loop between theoretical and empirical work in biology [51] (see also [43]).

Given these statistics, it is worth asking whether biology currently suffers from a theory crisis and, if so, how it might be remedied. Our aims are four-fold. First, we consider communication breakdowns between theoreticians and empiricists through the lens of a theory crisis to illustrate how such breakdowns could contribute to both research waste and the broad-sense replication crisis. Next, we review proposed solutions for issues associated with the theory crisis, including theory-empirical communication failures from the social and biological sciences. We then highlight a critical gap in a scientific cycle — the lack of systematic mapping of theoretical models — which, when addressed, can facilitate communication between theoreticians and empiricists. Finally, we shift our focus to two issues: the low numbers of pure theoreticians in biology and their diversity with respect to identity, geography, and academic training. We describe two solutions to ameliorate these issues, which could help not only create diverse science teams between theoreticians and empiricists but also turn more people into theoreticians and liaisons between the two groups.

In this essay, our examples will primarily come from ecology and evolutionary biology because they are our primary fields. Also, ecology and evolutionary biology are fields rich in formal theory, making them suitable for illustrating our points, although the literature suggests theory-empiricism communication breakdowns in biology, including neuroscience, molecular biology, and biomedical sciences [53–56]. Yet, the issues we discuss are relevant to biology as a whole, and the proposed solutions can be adapted to other disciplines facing similar challenges.

## Theory before method: questionable research practices and research waste in relation to theory

While the replication crisis (narrow-sense) is often attributed to methodological or reporting shortcomings in data collection and analysis, the theory crisis stems from misunderstanding, miscommunication, or misapplication of theory—an epistemic failure rather than a methodological one [37, 38]. In biology, as in psychology, researchers heavily rely on inferential statistics, especially *null hypothesis significance testing (NHST)* [57, 58]. In doing so, researchers often derive their hypotheses or predictions from existing theory, forming the alternative hypothesis within the NHST framework. Then, they statistically assess their hypothesis, using methods such as *t*-tests or generalized linear mixed-effects models to test and potentially reject the null hypothesis.

In psychology, several authors have recently pointed out that this statistical translation of theory is problematic because many theories and hypotheses are verbal, so interpretations of the theories can become unconstrained or subjective and, therefore, ungeneralizable [34, 36–40]. In biology, while similar issues can occur, many theories are based on precise mathematical logic or computer simulation, providing *directional* or *quantitative predictions* that are then tested in nature (e.g., [45]). However, mathematical formalization alone does not always lead to the development of stronger hypotheses. Even when theoretical predictions are clear, such predictions often depend on parameters that can vary widely and produce different or opposing predictions. These parameters may be difficult or impossible to measure accurately or precisely in empirical systems. As a result, empiricists may struggle to apply the theory appropriately, leading them to use approximations or make subjective choices to fit what they can test into the theoretical framework. This process can lead to misinterpretation or misapplication of formal theories [47] because empiricists might inadvertently oversimplify complex models or ignore critical parameter dependencies.

An example illustrating challenges arising from the oversimplification of complex theories comes from modern coexistence theory (MCT) [59, 60]. As pointed out by Terry and Armitage [61], MCT is less a predictive, testable theory but rather an analytical framework for partitioning growth rates into various coexistence-promoting mechanisms. Nonetheless, MCT has advanced community ecology by concentrating numerous system- and model-dependent theories of species coexistence under a small, shared set of mechanisms. However, this deceptively simple framework, where biological concepts such as “niche differences” are assigned numerical indices, is at risk of being superficially applied by empiricists, since the results from an MCT-based analysis are only as robust as the quality of one’s collected data, choice of underlying model, and fitted parameter estimates. This has led to most hypotheses in MCT studies being quite nondirectional and qualitative, with predictions that are difficult to externally validate and generalise [61].

Importantly, theories that produce ambiguous predictions are not inherently poor; they often reflect the complexity of biological systems where multiple processes interact. Using such theories to guide empirical research is valuable but requires careful consideration of all plausible outcomes and understanding the conditions under which different predictions might hold. When researchers formulate hypotheses without fully engaging with the theory’s complexities, they may test irrelevant or oversimplified hypotheses, leading to research waste. Such barriers are not unique to community ecology but occur in all fields where training in writing and interpreting formal theory is not a central theme in the scientific curriculum [49].

Turning to evolutionary biology, our following example concerns the “extrinsic mortality hypothesis,” proposed in 1957 by G. C. Williams [62]. This nonformal, verbal theory suggested that high extrinsic mortality rates would select for increased growth rates, reproduction, and aging, in turn leading to shorter maximum lifespans (reviewed in [63]; see also [64–68]; note that this example is similar to cases in psychology when nonformal theories motivate empirical studies). However, subsequent formal analyses demonstrated that, depending on the underlying assumptions and trade-offs, extrinsic mortality could either increase or decrease the rate of aging or be unrelated altogether (summarized in [69]); therefore, previous studies were often misdirected because these studies might have studied systems where Williams’ original verbal predictions did not apply. This example demonstrates the importance of the formal model. Also, it is critical to understand such formal models, and which set of assumptions (or parameter ranges) is most relevant to one’s study system to make more accurate and relevant predictions.

Based on these examples, we suggest that a theory crisis can lead to three types of questionable research practices, QRPs (see Fig. 1). These three “new” extensions of QRPs are counterparts to well-known issues of empirical studies: HARKing (hypothesizing after results are known), ***p*-**hacking (or data dredging), and cherry-picking (of results to report).

The first is called *H-BUTing* (/hei-tʃ-but-ing/) or “Hypothesizing Before Understanding Theory.” H-BUTing occurs when researchers formulate hypotheses without fully understanding the underlying theory, especially when the theory produces ambiguous or context-dependent predictions. H-BUTing may often happen because the theory is complex, involves multiple interacting mechanisms, or depends on parameters that are difficult to measure, as mentioned above. Also, H-BUTing can be equated to the previously identified issue of “premature hypothesis testing” [39]. It is premature because researchers either do not understand a theory or do not understand if their study systems are suitable for testing the theory.

We call the second QRP hypothesis hacking or *h*-hacking; *h*-hacking involves deliberately manipulating or selectively interpreting theories to generate hypotheses that align with preconceived beliefs or are more likely to yield significant results. Researchers might emphasize certain mechanisms within a theory while ignoring others or adjust theoretical assumptions to produce a desired prediction. Also, generating surprising (highly unrealistic) hypotheses can be a part of *h*-hacking because supporting a surprising hypothesis can lead to a high-profile journal publication (cf. [70]; see also [71]).

The third is “hypothesis cherry-picking” (termed by Krämer [34]), where researchers deliberately ignore alternative, theoretically, valid hypotheses that do not support their initial beliefs or the most favorable interpretation of their results. Such selective use of hypotheses theories goes against the concept of strong inference, where multiple plausible hypotheses are considered and tested in a study [72].

All these “new” QRPs are different from the more recognised QPRs, as H-BUTing, *h*-hacking and hypothesis cherry-picking occur before data collection while HARKing *p*-hacking and selective reporting (cherry-picking) occur afterwards. We note that H-BUTing and *h*-hacking are similar and indeed related, yet they are different, as the former is unintentional while the latter is deliberate. Regarding intentionality, *h*-hacking (deliberate misinterpretation) and hypothesis cherry-picking (deliberate negligence of alternatives) are alike.

In addition to these theory-related QRPs, we can add “poor conceptual design” alongside poor methodological design, publication bias, and incomplete reporting as the primary contributors to research waste. Epistemological issues (i.e., conceptualisation issues) lie upstream of methodological issues in the design and conduct of research (Fig. 1). Therefore, solving downstream issues, such as incomplete reporting, does not fix upstream issues, such as improper use of theory. Therefore, we believe that solving upstream poor conceptual design, a part of the theory crisis, is more critical than other downstream methodological issues pertaining to the (narrow-sense) replication crisis.

Notably, if poor conceptualization results in inappropriate experimental design or measurement, it can compromise a study’s *construct validity* and *internal validity* (for difficulties in measuring theoretical constraints, see [73]). At the same time, poor conceptualization can also threaten *external validity* (generalizability), for example, through the biases in the selection of study systems, as a certain theory is more easily tested in particular organisms or locations [40] (for reflection on this issue, see “Western, Educated, Industrial, Rich, and Democracies”, WEIRD [74] and “Social background, Trappability, Rearing history, Acclimation and habituation, Natural changes in responsiveness, Genetic make-up, and Experience”, STRANGE [75]).

## Proposed solutions: more development, education, and collaboration

Proposed solutions can be grouped into three initiatives. First, researchers can do more work to understand and operationalize theory before conducting a study with hypothesis testing (also referred to as a confirmatory study). Scheel and colleagues argue that premature hypothesis testing is rampant in psychology, but researchers should conduct more *non-confirmatory studies* (sometimes referred to as “exploratory” or “discovery-oriented” studies; [38], cf. [39]). Non-confirmatory studies can resolve issues of H-BUTing (hypothesizing before understanding theory) because they will identify whether and when a model is relevant to their biological system by understanding model assumptions and parameter space (e.g., a model assuming semelparous organisms without non-overlapping generations) [39].

For example, an ecologist might use a toy theoretical model to investigate how differences in the concentration of a shared resource and the uptake rates by competing species influence their coexistence, before designing experiments to test specific hypotheses. Such exploratory analyses can reveal conditions under which certain hypotheses concerning shifts in coexistence are valid, guiding more effective confirmatory studies and leading to more directional, quantitative hypotheses tailored to the specific system under study. A non-confirmatory exploration is often warranted before embarking on confirmatory work, especially at the start of postgraduate research programs. An alternative approach we find valuable is reproducing a theoretical study’s results on our own (typically on a computer). Once this is accomplished, the models can be further modified and played with to more thoroughly understand their scope and assumptions.

However, we acknowledge that current academic incentives often favor confirmatory studies with clear, significant results [76]. Non-confirmatory or exploratory studies may be perceived as less valuable or publishable, which can discourage researchers from undertaking them. To encourage more non-confirmatory research, it is crucial to adjust incentive structures within academia and publishing. Journals and funding agencies can play a role by recognizing the importance of exploratory studies in advancing theoretical understanding and promoting policies that value such contributions more. Also, from our experience, publishing such a non-confirmatory study is often an important precursor, as discussed above, to publishing an important confirmatory work, so this process itself can act as an incentive for conducting and publishing non-confirmatory studies.

Second, universities can offer undergraduate and postgraduate courses on how to understand theoretical and mathematical/computational models and, more broadly, the role of theory in the scientific process, thereby promoting the training of pure theoreticians and theoretically proficient empiricists (i.e., liaisons). Some psychologists have shared their experiences on the effectiveness of such courses [35, 41] (see also [43]). For example, Borsboom, and van der Maas teach a ‘theory construction’ course where, in addition to lectures on the methodology, students create computer simulation models that can explain a phenomenon well but also see how a model is limited as it creates non-sensical values under some settings [35]. We also think such a hands-on course would be eye-opening and useful training.

In the biological sciences, however, theoreticians are almost always a minority group who teach courses that a minority of students attend, and often at levels more advanced than the average biosciences student is proficient. Thus, although education is essential and effective, it cannot break the status quo of early separation between theoreticians (who often arrive at biology with math or physics degrees) and empiricists (who tend to avoid most math and physics classes beyond prerequisites). This is also because understanding math takes time, and interventions should start much earlier than university education. Furthermore, we have noticed a concerning trend in ecology and evolution, where advertisements for pure theoreticians seem to be declining. In contrast, appointments in “quantitative biology” are on the rise. We speculate that this may reflect a growing trend of interest in the more lucrative fields of data science and bioinformatics, which compete with the theoretical sciences over a shared pool of students. Nevertheless, online education and technologies can overcome the shortage of theoreticians (more discussed later).

Third, theoreticians and empiricists should collaborate more. Recently, Ou and colleagues have described how theoreticians can write more accessible papers by being mindful of what parts of their analyses are the most difficult for empiricists to understand [44]. Likewise, Grainger and colleagues have produced an excellent guide for empiricists with tips on understanding and testing formal theory [43]. Notably, these two papers largely overlap in their author lists, which comprise a mixture of empiricists, theoreticians, and liaisons in ecology and evolutionary biology. Though the primary means by which theoreticians and empiricists interact is indirectly through citation of one another’s publications, direct collaboration might be a more effective means for reducing barriers in understanding, though the relative scarcity of theoreticians in most biological fields can limit such direct interactions.

Interestingly, a group of psychologists have successfully attempted an exercise called the “Many Modelers Hackathon” where a few theoreticians worked with many empiricists to formalize verbal theories during a 3-h workshop [77]. This Many Modelers event was successfully run as part of a 2021 conference for the Society for the Improvement of Psychological Science (SIPS)—a model for the Society for Open, Reliable, and Transparent Ecology and Evolutionary Biology (SORTEE). A Many-Modelers Hackathon holds tremendous potential in biology, which has a long history of formalizing theories and designing complex experiments (more on the potential of this type of *hackathon* later).

## A gap in the scientific cycle and a proposed solution: systematic mapping of theoretical models

**Fig. 2:**
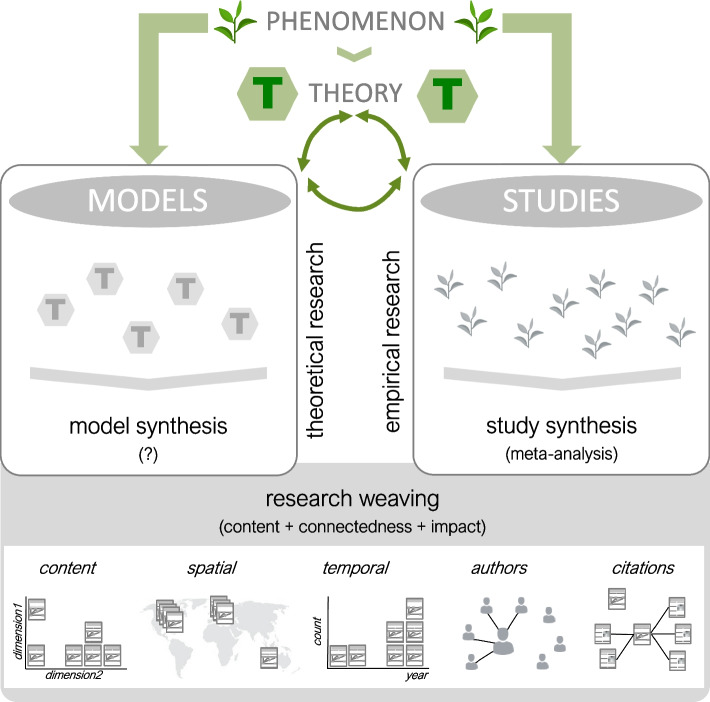
A depiction of a scientific cycle. Researchers seek to understand a phenomenon and develop a theory while engaging with theoretical research or empirical research. While meta-analysis has revolutionized empirical synthesis, the synthesis of theories (models) is primarily narrative. Research weaving (systematic mapping and bibliometrics) could help not only synthesize theoretical models but also summarize both types of research on a topic

In Fig. 2, we illustrate a scientific cycle where researchers seek to understand a phenomenon and develop a theory via two pathways: empirical research and theoretical development. The last three decades have seen a revolution in empirical research with a rise in research synthesis, especially meta-analysis [78, 79]. *Meta-analyses* (often a part of *systematic reviews*) have gradually been superseding traditional narrative reviews because meta-analyses can bring about unique benefits that are not possible with narrative reviews [80]. For example, meta-analyses can quantitively identify general patterns and knowledge gaps, explain inconsistencies among empirical studies, and generate ideas that can fledge into theories [81].

Unlike meta-analyses, the synthesis of theory is primarily narrative rather than systematic, representing a gap in the scientific cycle (Fig. 2) [82] (cf. [83]). While conducting a quantitative synthesis of theoretical literature may be challenging (cf. [84]), a systematic qualitative synthesis is still possible. Such syntheses might encompass a systematic review or a systematic map (sometimes, known as a scoping review; see [85, 86]). Although often mixed up, systematic reviews and *systematic maps* have different objectives [87, 88]. The former answers a specific question, often relating to “What works?” (e.g., does an intervention have an effect?), while the latter addresses a more general question, such as “What has already been studied and where do gaps remain?” [89]. Therefore, a systematic map is a structured collection of studies, making it suitable for qualitatively summarizing models. Systematic maps can reveal general patterns and knowledge gaps in related models. Additionally, mapping models can identify gaps in empirical observations by examining how parameter values in theoretical models are defined, whether based on empirical results or assumptions requiring further validation. Although their number is limited and often (mis)labeled as a systematic review, systematic maps of models exist in various fields of medicine and biology.

One noteworthy systematic map has been conducted for the field of life history theory by Mathot and Frankenhuis [90]. Many empiricists have tested hypotheses concerning covariation between life history traits within species such as growth rate and age of reproduction with behavioral and physiological traits, theorized to arise from trade-offs between investment in current and future reproduction, known as “pace-of-life syndromes” [91]. Here, the authors attempted to map theoretical models on this topic, but they only identified two unique models [90]. Importantly, these models provided mechanisms by which covariances could arise between life history and other traits without invoking the current-future reproduction trade-offs. This finding suggested that many empirical studies may have tested hypotheses lacking comprehensive theoretical support, potentially committing H-BUTing and hypothesis cherry-picking (Fig. 1). Here, the systematic mapping revealed a lack of formal theoretical models, and identified ways in which future theoretical development will best allow for their empirical evaluation given their particular assumptions.

This example might give the impression that such mapping activities are futile due to the paucity of theoretical papers in some research areas in biology. However, certain topics enjoy an abundance of theory. A systematic map of theory relating to antimicrobial resistance, for example, identified 273 studies of mathematical models made at the population level [92] (see also [93]); this map elucidated theoretical gaps, such as the scarcity of models on transmission between humans and animals and the consideration of environmental factors. Similarly, another systematic map collated 698 studies of agent-based models of infectious disease transmission published from 2006 to 2015 [94].

Recently, Achter and colleagues have published an opinion article promoting systematic maps (and reviews) of agent-based models so that new models do not “re-invent the wheel” but rather build upon previous models in environmental sciences [82]. They argue that such a systematic map can lead to further refinement and the development of a theory. We concur and join their call for systematic maps of mathematical and computational models. Importantly, systematically mapping models could guard against hypothesis cherry-picking and *h*-hacking (Fig. 1). This is because a map gives a comprehensive catalog of current theoretical models, which can direct the development of a set of testable hypotheses, hopefully reducing the incidence of cherry-picking hypotheses or liberal interpretations of theories.

Recently, bibliometric analyses in the forms of quantifying articles’ citation impacts and connections through citations or collaborations have been conducted for models of antibiotic resistance [95] in addition to the systematic map mentioned above [92]. Some of us (SN and ML) have recently proposed a new way of synthesizing literature, named *research weaving*, which combines systematic mapping and bibliometrics [89]. As outlined above, systematic mapping summarizes the current state of knowledge and evidence, identifying areas with research gaps or an abundance of papers. As a complementary approach, bibliometrics enables researchers to see how pieces of evidence are connected. Such analysis can reveal the structure and development of a field and how influential particular works have been (research weaving examples, see [96, 97]). Therefore, a research weaving of theoretical models holds much potential, for example, for collaboration work between theoreticians and empiricists because both can contribute to such an activity (cf. [82]) and can lead to the synthesis of both theoretical and empirical results (Fig. 2).

Furthermore, some bibliometric analyses (i.e., geographic and collaboration-network analyses) can reveal the lack of inclusion, diversity, equity, and access (IDEA; also referred to as EDI or DEI) in a field of study by examining geographic distribution and collaboration networks. Identifying underrepresented regions or groups can help target efforts to promote diversity and inclusivity in theoretical biology. Enhancing IDEA can help ameliorate the theory crisis by bringing diverse perspectives and expertise, which we outline in the next section.

## Ideas to create a tight theory-empirical feedback loop by achieving IDEA: inclusion, diversity, equity, and access

Several articles have pointed out the shortfall of IDEA in the theoretical biology community [43, 98, 99]. Evidence suggests theoreticians are an impactful yet privileged group lacking diversity (e.g., [100]). This lack of diversity may partly stem from the smaller size of the theoretical community or its concentration primarily at top institutes in wealthy nations (c.f., [101]). Increasing the pool of theoreticians with IDEA goals in mind could ameliorate both the theory crisis and the lack of diversity by bringing in new perspectives and fostering innovation in theoretical development. In recent years, many academic societies in biology have initiated efforts to promote IDEA by encouraging historically underrepresented and marginalized groups to join and take on leadership roles, achieving noticeable success (cf. [102]).

Theoretical work is impactful and provides mathematical and computational skills that are highly transferable. It also offers flexibility, as theory development can often be conducted with minimal resources—a pen and paper or a laptop—making it accessible to researchers from institutions with limited funding for large lab or field-based programs. By promoting theoretical training among diverse groups, we can help break down academic inequities and empower researchers globally. Additionally, addressing the scarcity of theoreticians is urgent because researchers now regularly collect and analyze massive, complex datasets with only a small pool of theories to generate a priori hypotheses (e.g., [103]). The overabundance of data highlights the importance of theory and theoreticians in advancing scientific understanding.

To achieve these goals of growing and diversifying the community of theoretical biologists, we propose two strategies. First, academic societies can create free, accessible training resources on understanding and building theoretical models. For example, online video series can democratize knowledge, similar to Richard McElreath’s “Statistical Rethinking” lectures, which make Bayesian statistics and causal inference accessible to biologists [104]. Additionally, initiatives like the International Initiative for Theoretical Ecology’s online seminar series have successfully attracted a global audience (https://iite.info/seminar/). These resources can help cultivate theoretical skills among researchers from diverse backgrounds and regions.

Second, societies can incorporate collaborative activities like hackathons into their conferences, fostering direct interaction between theoreticians and empiricists. Events such as Many-Modelers hackathons [77] demonstrate how such collaborations can formalize verbal theories and generate testable hypotheses. It is important to note that programming skills are not a prerequisite for participating in a hackathon; the term now widely refers to any event structured around team-based creative problem-solving. For example, assembling ecology and evolution journal editors to develop shared guidelines for accepting registered reports is a form of a hackathon that leads to impactful outcomes [105–107]. Along with hackathons, conferences could include unstructured discussion events where participants are prompted to discuss ideas without a pre-specified task (called *unconference*). These activities promote inclusivity by bringing together researchers from diverse backgrounds and career stages, fostering new collaborations and enhancing theoretical understanding. One example of this type of unstructured conference in ecology is the annual “ANdiNA” meeting held to “align global priorities and local action in ecological research and practice.”

We (SN and ML) have organized several hackathons and unconferences at SORTEE annual online meetings; one of the activities has already resulted in a publication [108] (other examples of SORTEE conference publications [109–111]). These events have facilitated team science by encouraging interdisciplinary work and appropriately acknowledging individual contributions, such as by developing the “Methods Reporting with Initials for Transparency” (MeRIT) system [108]. By embracing IDEA principles in these ways, we can create a more robust and diverse theoretical community. This process, in turn, strengthens the theory-empirical feedback loop, promoting the development of well-founded theories and enhancing the integration of theory and empirical research across biology.

## Less waste and more IDEA for the future of biology

**Fig. 3:**
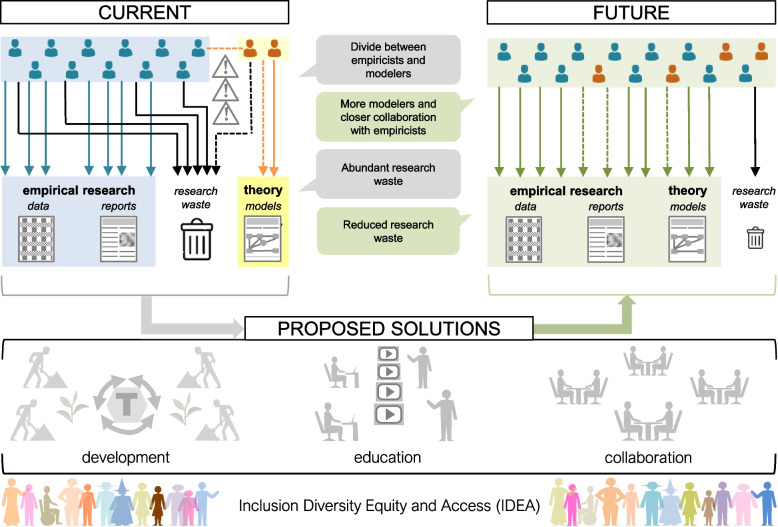
The current and future of empirical and theoretical research. Currently, due to miscommunications between theoreticians (minority) and empiricists (majority), resulting in research waste. Via the proposed solutions, development, education, and collaboration, the future research community will have more theoreticians working with empiricists, especially if learned societies can embrace the theory crisis and promote the integration of theoretical and empirical work through IDEA. Solid lines represent no direct collaborations, while dotted lines indicate direct collaborations (the upper panels). While research waste may be an unavoidable part of the scientific process, more research efficacy can be attainable

Here, we have described how biology may be prone to a theory crisis, which contributes to a replication crisis (broad-sense) and research waste. We have also discussed existing and potential solutions—such as the systematic mapping of theoretical models—to help resolve the theory crisis (Fig. 3). Despite past attempts to fix theory-empirical communication breakdowns, dramatic success has yet to be achieved in creating a tight feedback loop between theory and experimentation (cf. [43]). We contend that pursuing IDEA can, at least partially, ameliorate such breakdowns and presented two concrete ideas—engaging educational videos and Many-Modelers-like-Hackathon events at conferences—that academic societies are encouraged to act upon.

We encourage you and your scientific community to generate more ideas and mobilize these ideas to improve both theory-empirical communication and IDEA. By fostering a diverse team science approach, we can tackle both big questions in biology and pressing ecological and environmental issues that humanity faces. We believe that embracing these strategies will lead to a future where research waste is minimized, and the integration of theory and empiricism is strengthened, ultimately advancing the field of biology.

## Data Availability

No datasets were generated or analysed during the current study.
